# The effect of pregnancy-related hormones on hepatic transporters: studies with premenopausal human hepatocytes

**DOI:** 10.3389/fphar.2024.1440010

**Published:** 2024-08-07

**Authors:** Jhohann Richard de Lima Benzi, Yik Pui Tsang, Jashvant D. Unadkat

**Affiliations:** Department of Pharmaceutics, School of Pharmacy, University of Washington, Seattle, WA, United States

**Keywords:** pregnancy hormones, hepatic transporters, transporter mRNA expression, transporter activity, plated human hepatocytes, transporter-mediated hepatic drug clearance

## Abstract

**Introduction:**

Pregnancy results in significant changes in drug pharmacokinetics (PK). While previous studies have elucidated the impact of pregnancy-related hormones (PRH) on mRNA or protein expression and activity of major hepatic metabolizing enzymes, their effect on hepatic drug transporters remains largely unexplored. Therefore, we investigated the effect of a cocktail of PRH on the mRNA expression and activity of hepatic transporters.

**Methods:**

Plated human hepatocytes (PHH) from 3 premenopausal donors were incubated, in triplicate, for 72 h, with vehicle (DMSO < 0.01%), rifampin (10 μM; positive control) or a cocktail of PRH consisting of estrone, estradiol, estriol, estetrol, progesterone, cortisol, testosterone, oxytocin, and placental growth hormone. The PRH concentrations replicated 0.1×, 1×, or 10× of the plasma concentrations of these hormones observed during each of the three trimesters of pregnancy. After treatment, mRNA expression (quantified by qPCR) of hepatic influx and efflux transporters as well as the activity of influx transporters was quantified (uptake of a selective substrate ± corresponding transporter inhibitor). The data were expressed relative to that in the control (vehicle) group. Significance was evaluated by ANOVA (followed by Dunn’s multiple comparisons) or unpaired *t*-test when the within-lot data were analyzed, or repeated measures ANOVA (followed by Dunn’s multiple comparisons) or paired *t*-test when data from all 3 lots were analyzed (*p* < 0.05).

**Results and Discussion:**

In general, a) PRH cocktails significantly induced transporter mRNA expression in the following order OAT2 ≈ NTCP ≈ OCT1 > OATP2B1 and repressed mRNA expression in the following order OATP1B3 > OATP1B1; b) these changes translated into significant induction of OAT2 (T1-T3) and NTCP (T2-T3, in only two lots) activity at the 1× PRH concentration. Compared with the influx transporters, the induction of mRNA expression of efflux transporters was modest, with mRNA expression of MRP2 and BSEP being induced the most.

**Conclusion:**

Once these data are verified through *in vivo* probe drug PK studies in pregnancy, they can be populated into physiologically based pharmacokinetic (PBPK) models to predict, for all trimesters of pregnancy, transporter-mediated clearance of any drug that is a substrate of the affected transporters.

## 1 Introduction

According to the CDC (Centers for Disease Control and Prevention) and others, 68%–90% of pregnant people take medication during pregnancy (Mitchell et al., 2011; Centers for Disease Control and Prevention, 2023; Werler et al., 2023). They do so to treat pre-existing conditions or to manage illnesses arising during or because of pregnancy (Mitchell et al., 2011; Werler et al., 2023). However, over 90% of clinically approved drugs prescribed to pregnant people are taken “off-label,” i.e., without information on their pharmacokinetics (PK), safety, and efficacy during pregnancy (Scaffidi et al., 2017). Pregnancy can change the PK of drugs by repressing or inducing drug transporters or metabolizing enzymes (Ke et al., 2014; Coppola et al., 2022) resulting in drug toxicity or lack of efficacy, respectively. For example, exposure (plasma area under the curve, AUC) to the antiretroviral indinavir is reduced in pregnant people by 68% during the third trimester vs. *postpartum* period, resulting in subtherapeutic plasma trough concentration (Unadkat et al., 2007). Decreased exposure to buprenorphine and nifedipine during the second and third trimester requires adjustments in their dosing regimens to manage opioid withdrawal and hypertension, respectively (Caritis et al., 2017; Mulrenin et al., 2021).

It is neither feasible nor desirable to determine the pregnancy-induced changes in PK of all drugs taken by pregnant people. Therefore, an alternative and appealing systems pharmacology approach has been adopted by many investigators, including us. That is, to conduct *in vivo* phenotyping studies with drugs that are predominately cleared from the body by a single hepatic transporter or metabolizing enzyme. For example, midazolam and digoxin were used as phenotyping drugs to report the *in vivo* changes in CYP3A and P-gp activity, respectively, in the third trimester (Hebert et al., 2008). Once the change in hepatic enzyme/transporter activity due to pregnancy is quantified, it can be applied to predict the disposition of other drugs, during pregnancy, that are also cleared by the same enzyme/transporter (in part or in whole). Therefore, such phenotyping studies allow extrapolation to many drugs. While this approach has been highly successful for hepatic CYP enzymes (Coppola et al., 2023), it has not been widely used to quantify the changes in hepatic transporter activity during pregnancy. This is because phenotyping probes for hepatic transporters are not selective as they are often transported by multiple transporters (e.g., OATPs). And, even if such probes were available, conducting *in vivo* phenotyping studies in pregnant people through different stages of pregnancy is logistically and ethically challenging. For these reasons, phenotyping studies, even for hepatic CYP enzymes, are mostly limited to the third trimester. Therefore, alternative approaches that can predict the PK of drugs during different gestational ages are needed.

One alternative approach is to quantify the induction or repression of hepatic transporters and enzymes in human hepatocytes exposed to pregnancy-related hormones (PRH) at plasma concentrations observed during the three trimesters (T1-T3) of pregnancy. Then, these data can be used, through physiologically based PK modeling, to predict the disposition of drug substrates of hepatic transporters during pregnancy. PRH such as estrogens, progesterone, cortisol, and growth hormones are known regulators of hepatic CYP enzyme activity and expression (Dickmann and Isoherranen, 2013; Isoherranen and Thummel, 2013; Papageorgiou et al., 2013; Khatri et al., 2021a; Khatri et al., 2021b; Brouwer et al., 2022). Therefore, the primary objectives of this study were 1) to quantify the effect of a cocktail of PRH on the mRNA expression of major influx (e.g., OATPs, OCT1, OAT2, and NTCP) and efflux (e.g., P-gp, BCRP, MRP2-4 and BSEP) hepatic transporters in female premenopausal plated human hepatocytes (PHH); 2) to determine if changes in transporter mRNA expression translate to changes in the activity of these transporters. Only changes in influx transporter activity were measured as canalicular efflux transporter activity is best measured using sandwich-cultured hepatocytes. The PHH were incubated with a cocktail of PRH consisting of estrone, estradiol, estriol, estetrol, progesterone, cortisol, testosterone, oxytocin, and placental growth hormone, all at concentrations that replicated 0.1×, 1× or 10× the plasma concentrations of these hormones observed during T1, T2, and T3. These concentrations mimicked the observed unbound (approximately), total, and supraphysiological plasma concentrations of PRH, respectively, in the three trimesters.

## 2 Materials and methods

### 2.1 Reagents and chemicals

The PRH estrone (E1), estradiol (E2), estriol (E3), estetrol (E4), progesterone, testosterone, oxytocin, the drug rifampin, and dimethyl sulfoxide (DMSO) were obtained from Sigma-Aldrich (St. Louis, MO, United States). The placental growth hormone (PGH) was obtained from R&D Systems (Minneapolis, MN, United States) and cortisol was obtained from Merck (Dortmund, Germany). Biocoat™ Collagen I coated Multiwell Plates were obtained from Corning (Bedford, MA, United States). Enzyme-linked immunosorbent assay (ELISA) kits for oxytocin determination were purchased from Cayman Chemical (Ann Arbor, MI, United States). PureLink™ RNA Mini Kit (Invitrogen, MA, United States), and High-Capacity RNA-to-cDNA™ Kit were obtained from Applied Biosystems (Carlsbad, CA, United States). Pierce™ BCA Protein Assay Kit, Universal PCR Master Mix, and TaqMan probes were purchased from Thermo Fisher Scientific (Rockford, IL, United States). The TaqMan probes employed were: OATP1B1 (Hs00272374_m1), OATP1B3 (Hs00251986_m1), OATP2B1 (Hs01030343_m1), OAT2 (Hs00198527_m1), OCT1 (Hs01552829_m1), NTCP (Hs00161820_m1), BCRP (Hs01651967_m1), P-gp (Hs05638872_s1), MRP2 (Hs00960489_m1), MRP3 (Hs00978452_m1), MRP4 (Hs00988721_m1), BSEP (Hs00994811_m1) and GAPDH (Hs02786624_g1). Finally, the radionuclides [^3^H]-estradiol-17β-glucuronide, [^3^H]-estrone-3-sulfate, [^3^H]-cyclic guanosine monophosphate, [^3^H]-taurocholic acid and [^14^C]-metformin were purchased from American Radiolabeled Chemicals, Inc (St. Louis, MO, United States).

### 2.2 Culturing plated human hepatocytes (PHH)

Cryopreserved primary human plateable hepatocytes were purchased from or generously donated by BioIVT (Westbury, NY, United States). The hepatocytes were transporter-qualified, and the donors were all adult females of reproductive age (Table 1). Hepatocytes were plated and cultured as previously described by us (Papageorgiou et al., 2013). Briefly, the hepatocytes were thawed and plated in 96-well (for uptake experiments) or 24-well (for mRNA expression or oxytocin depletion experiments) collagen-coated plates at a density of 0.6 × 10^6^ human hepatocytes/mL of plating media. The PHH were incubated overnight at 37°C, 5% CO_2_, with INVITRO™ CP NPC Medium (BioIVT, Westbury, NY, United States) before conducting the experiments outlined below.

**TABLE 1:** Demographics of the female premenopausal hepatocyte donors.

Donor	Age	Race	Hispanic or latino	Cause of death	Alcohol use	Tobacco use	HIV and hepatitis B/C serology	Cytomegalovirus serology
ADR	38	Caucasian	No	Trauma	No	No	Negative	Not reported
YND	37	Caucasian	No	Anoxia 2nd to SAH*	Yes	Yes	Negative	Positive
JEL	27	African American	No	Anoxia	No	No	Negative	Positive

SAH, Subarachnoid hemorrhage.

### 2.3 Incubation of PHH with PRH after adjusting their concentrations due to depletion by the hepatocytes

PHH were incubated in triplicate for 72 h, at 37°C in 5% CO_2_, with the PRH, rifampin (10 µM), or vehicle (DMSO <0.01%) in a custom-made INVITROGRO™ HI Medium (BioIVT, Westbury, NY, United States) devoid of dexamethasone and cortisol. The medium was refreshed daily (also see below for details on refreshment of medium containing PRH). Separate wells were designated for mRNA expression and activity experiments. Rifampin served as a positive biological control as it is a potent and well-studied inducer of hepatic CYP3A4 mRNA expression.

The nominal PRH concentrations were based on the geometric mean of the observed PRH plasma concentrations in each trimester (Table 2). The trimesters were defined according to the American College of Obstetricians and Gynecologists classification (The American College of Obstetricians and Gynecologists, 2024). Then, the PRH nominal concentrations were adjusted to account for their depletion by PHH in culture. The rate constants for depletion of estrogens (E1-E4), progesterone and testosterone were obtained from the literature (Li, 2008; Choi et al., 2013; Zhang et al., 2015; Fashe et al., 2022). Cortisol and PGH are not depleted by human hepatocytes (Zhang et al., 2015; Fashe et al., 2022). The depletion of oxytocin by PHH was assessed in-house as follows. Briefly, the 3 lots of PHH were incubated, in triplicate, with oxytocin as a part of the PRH cocktail at 1× and 10× T3 concentrations (Table 2) for 72 h with daily refreshment of the medium (containing the PRH). During the final 24 h, 3−4 aliquots (50 µL each) were collected at 0 (immediately after refreshing the medium) and after 3, 5, 6, 8, 10, and 24 h. The concentration of oxytocin in these samples was measured by ELISA. The elimination (or depletion) rate constant was estimated from log concentration vs. time data. The observed half-life of oxytocin in PHH was 150 ± 107 h, indicating that oxytocin was relatively stable when incubated with the PHH. Based on these PRH depletion half-lives, we adjusted the actual PRH concentrations used in the medium (Table 3) such that the area under the curve of the hormone concentration during the hepatocyte incubation time (AUC/τ) equaled the observed steady-state plasma concentration of the hormone during each trimester (Table 2); τ denotes the time (8 and 16 h) when medium was refreshed daily.

**TABLE 2:** Geometric mean of the reported steady-state total plasma concentrations of PRH in each trimester (T1, T2, T3) of pregnancy.

PRH (ng/mL)	T1*	T2*	T3*	Source
E1^#^	0.80	4.42	9.75	De Hertogh et al. (1976), Caanen et al. (2016), Schock et al. (2016), Cohn et al. (2017)
E2^#^	1.01	6.14	13.97	De Hertogh et al. (1976), O’Leary et al. (1991), Soldin et al. (2005), Caanen et al. (2016), Schock et al. (2016), Cohn et al. (2017)
E3^#^	0.23	2.65	8.68	Loriaux et al. (1972), De Hertogh et al. (1976), Caanen et al. (2016), Cohn et al. (2017)
E4^#^	-	0.35	0.78	Coelingh Bennink et al. (2008a), Coelingh Bennink et al. (2008b)
Progesterone	26.31	51.66	140.11	Meulenberg and Hofman (1989), O’Leary et al. (1991), Soldin et al. (2005), Schock et al. (2016), Li et al. (2020)
Cortisol	143.54	269.82	312.36	Demey-ponsart et al. (1982), Soldin et al. (2005), Jung et al. (2011)
Testosterone	0.95	0.95	1.30	O’Leary et al. (1991), Caanen et al. (2016), Schock et al. (2016)
Oxytocin	0.18	0.23	0.23	Prevost et al. (2014), Uvnäs-Moberg et al. (2019)
PGH^#^	1.90	4.73	12.78	Wu et al. (2003), Chellakooty et al. (2004)

^*^T1, T2 and T3: first, second and third trimester of pregnancy; ^#^E1: estrone; E2: estradiol; E3: estriol; E4: estetrol; PGH: placental growth hormone.

**TABLE 3:** PHH were incubated for 72 h (medium was refreshed every 8 and 16 h daily) with PRH at the below-listed concentrations, adjusted for depletion where applicable.

PRH (ng/mL)	T1*		T2*		T3*	
	8 h	16 h	8 h	16 h	8 h	16 h
E1^#^	2.50	5.00	14.00	27.00	30.50	61.00
E2^#^	3.20	6.40	19.20	38.40	44.00	88.00
E3^#^	1.00	2.00	11.00	22.00	36.00	72.00
E4^#^	-	-	1.00	2.00	2.15	4.30
Progesterone	100.00	200.00	200.00	400.00	550.00	1,100.00
Cortisol^a^	150.0		270.00		320.00	
Testosterone	3.80	7.60	3.80	7.60	5.00	10.00
Oxytocin^a^	0.18		0.23		0.23	
PGH^a^	1.90		4.80		12.80	

*T1, T2 and T3: first, second and third trimester of pregnancy; ^#^E1: estrone; E2: estradiol; E3: estriol; E4: estetrol; PGH: placental growth hormone.

^a^ These hormones were not depleted by the hepatocytes. E4 is not produced in T1 (Coelingh Bennink et al., 2008a; Coelingh Bennink et al., 2008b).

### 2.4 Quantification of mRNA expression

After the PRH treatments, total RNA was isolated from the PHH using PureLink™ RNA Mini Kit following the manufacturer’s instructions. Total RNA was quantified using NanoDrop spectrophotometer 2000 (Thermo Fisher Scientific, Waltham, MA) and was reverse transcribed to cDNA using the High-Capacity cDNA Reverse Transcription Kit (Thermo Fisher Scientific, Waltham, MA) according to the manufacturer’s protocol. mRNA expression of key drug transporters was quantified by quantitative real-time PCR and expressed relative to GAPDH (housekeeping gene) using the 2^−ΔΔCt^ method. No C_t_ values lower than 33 were observed.

### 2.5 Quantification of influx transporter activity using selective transporter substrates

After the PRH treatment, PHH were washed twice with prewarmed Hank’s balanced salt solution (HBSS) buffer containing calcium and magnesium. Wells designed for NTCP activity determination were washed twice with prewarmed sodium-free buffer immediately before conducting the uptake experiments (Bi et al., 2017). The uptake of selective transporter substrates by the PHH was quantified (in triplicate), in the absence and presence of their corresponding inhibitor, over 15 min (a duration determined to be within the linear uptake range based on preliminary studies). All the selective substrates ± inhibitors and their concentrations were selected based on published literature: OATP1B1/3: 25 nM [^3^H]-estradiol-17β-glucuronide ± 200 µM bromsulfthalein (BSP), OATP2B1: 20 nM [^3^H]-estrone-3-sulfate +5 µM rifampicin (to inhibit OATP1B1/3) ± 200 µM BSP, OAT2: 80 nM [^3^H]-cyclic guanosine monophosphate ± 200 µM ketoprofen, OCT1: 10 µM [^14^C]-metformin ± 500 µM quinidine, and NTCP: 50 nM [^3^H]-taurocholic acid ± buffer containing sodium (Bi et al., 2017; Bi et al., 2019; Sachar et al., 2020). Sodium was removed from the uptake and wash buffers for NTCP inhibition by replacing NaCl and NaHCO_3_ with choline chloride and potassium bicarbonate, respectively, to maintain isotonicity. Since OATP1B1 and OATP1B3 activity cannot be distinguished, their combined activity was measured. Uptake was terminated by washing the PHH (3 times) with ice-cold HBSS or sodium-free ice-cold HBSS buffer (200 µL) (for NTCP uptake). Subsequently, 200 µL of 1 M NaOH was added to each well for cell lysis and incubated for 1 hour (at 37°C) followed by neutralization with 200 µL of 1 M HCl. For quantification, 300 µL of the lysate was mixed with 3 mL of Ecoscint A (National Diagnostics, Atlanta, GA) and subjected to liquid scintillation counting (PerkinElmer, Waltham, MA, United States). The amount of substrate taken up by the cells (in pmol) was normalized to the amount of total protein, which was determined by the bicinchoninic acid (BCA) assay using the Pierce BCA Protein Assay Kit (Thermo Fisher Scientific, Waltham, MA). The uptake values were normalized to the total protein determined using the BCA assay. The active uptake of each transporter was quantified by subtracting the passive uptake (with inhibitor or minus Na^+^) from the total uptake (without inhibitor or plus Na^+^).

### 2.6 Data analyses

Data analyses and descriptive statistics were performed using GraphPad Prism 10 (GraphPad Software, La Jolla, CA, United States). Data are presented as mean ± standard deviation unless otherwise stated. The mRNA expression of drug transporters and CYP3A4 as well as transporter activity was expressed relative to that in the control group. An ordinary one-way analysis of variance (ANOVA) followed by Dunn’s multiple comparison or unpaired *t*-test (where indicated) was performed when analyzing within-lot data, whereas repeated measures ANOVA followed by Dunn’s multiple comparison or paired *t*-test (where indicated) was performed when data from all 3 lots were analyzed. For each analysis, a p-value of <0.05 was considered statistically significant.

## 3 Results

**FIGURE 1:**
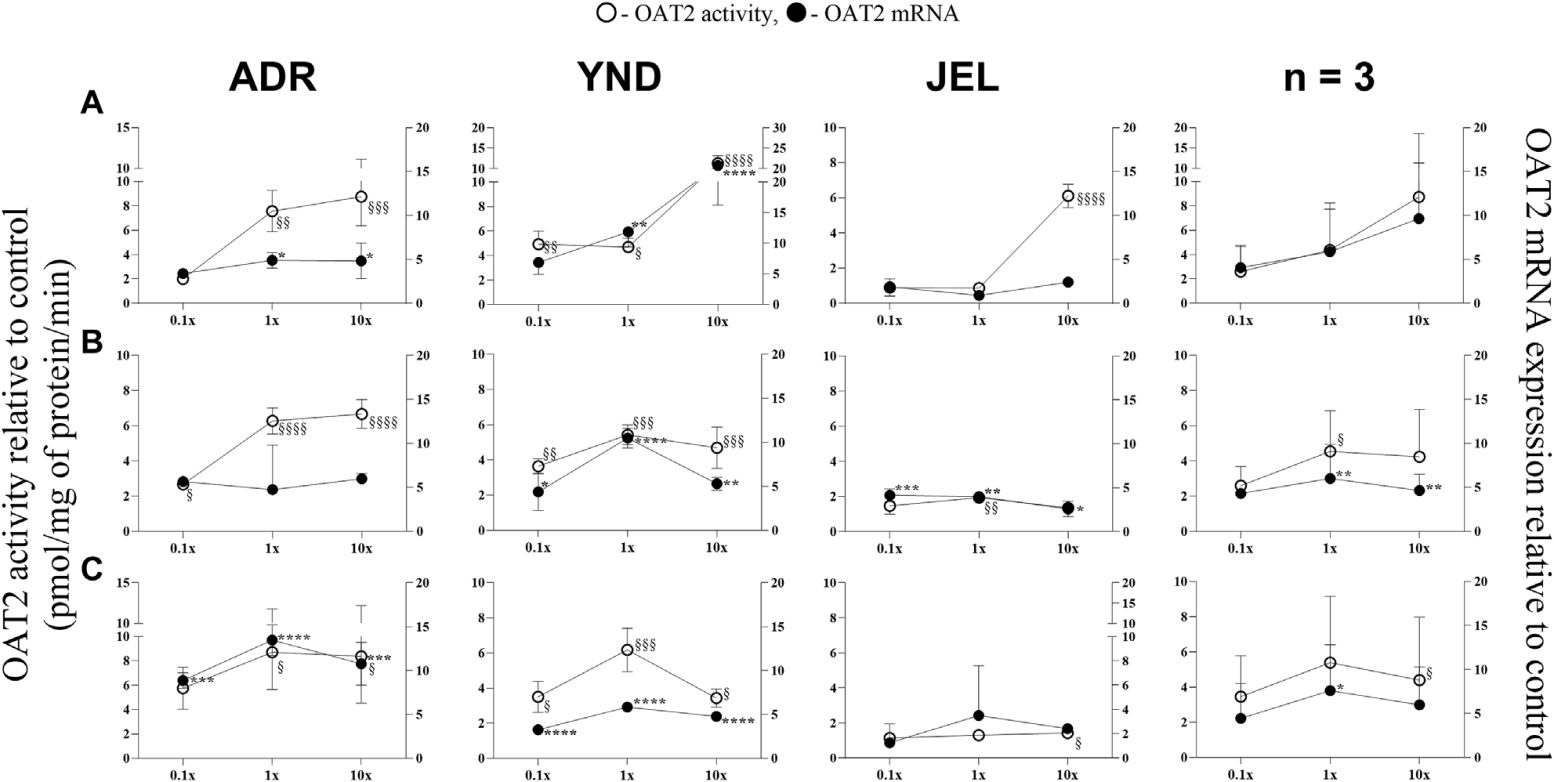
Effect (relative to control) of pregnancy-related hormone (PRH) cocktails on OAT2 activity (empty circles; left axis) and mRNA expression (filled circles, right axis) in each of the 3 lots of premenopausal plated human hepatocytes (ADR, YND, and JEL) as well as when these data were pooled (n = 3). PRH cocktails were designed to mimic their *in vivo* plasma concentrations observed in the first **(A)**, second **(B)**, and third **(C)** trimesters of pregnancy. Within each trimester, the effect of the ≈unbound (0.1×), total (1×), and supraphysiological (10×) plasma concentrations of the PRH was studied. Data are mean ± SD of 3 replicates for individual lots and mean ± SD of three lots when pooled. Significance was determined using one-way (within lot) or repeated measures (when data from 3 lots were pooled) ANOVA with Dunnett’s correction. For the mRNA data: *, *p* < 0.05; **, *p* < 0.01; ***, *p* < 0.001; ****, *p* < 0.0001; For the activity data: §, *p* < 0.05; §§, *p* < 0.01; §§§, *p* < 0.001; §§§§, *p* < 0.0001).

**FIGURE 2:**
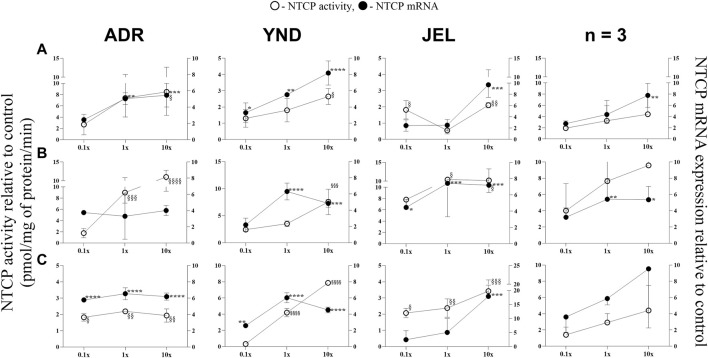
Effect (relative to control) of pregnancy-related hormone (PRH) cocktails on NTCP activity (empty circles; left axis) and mRNA expression (filled circles, right axis) in each of the 3 lots of premenopausal plated human hepatocytes (ADR, YND, and JEL) as well as when these data were pooled (n = 3). PRH cocktails were designed to mimic their *in vivo* plasma concentrations observed in the first **(A)**, second **(B)**, and third **(C)** trimesters of pregnancy. Within each trimester, the effect of the ≈unbound (0.1×), total (1x), and supraphysiological (10×) plasma concentrations of the PRH was studied. Data are mean ± SD of 3 replicates for individual lots and mean ± SD of three lots when pooled. Significance was determined using one-way (within lot) or repeated measures (when data from 3 lots were pooled) ANOVA with Dunnett’s correction. For the mRNA data: *, *p* < 0.05; **, *p* < 0.01; ***, *p* < 0.001; ****, *p* < 0.0001; For the activity data: §, *p* < 0.05; §§, *p* < 0.01; §§§, *p* < 0.001; §§§§, *p* < 0.0001).

**FIGURE 3:**
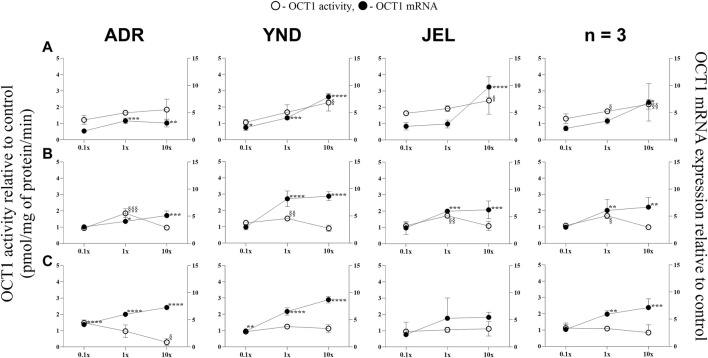
Effect (relative to control) of pregnancy-related hormone (PRH) cocktails on OCT1 activity (empty circles; left axis) and mRNA expression (filled circles, right axis) in each of the 3 lots of premenopausal plated human hepatocytes (ADR, YND, and JEL) as well as when these data were pooled (n = 3). PRH cocktails were designed to mimic their *in vivo* plasma concentrations observed in the first **(A)**, second **(B)**, and third **(C)** trimesters of pregnancy. Within each trimester, the effect of the ≈unbound (0.1×), total (1x), and supraphysiological (10x) plasma concentrations of the PRH was studied. Data are mean ± SD of 3 replicates for individual lots and mean ± SD of three lots when pooled. Significance was determined using one-way (within lot) or repeated measures (when data from 3 lots were pooled) ANOVA with Dunnett’s correction. For the mRNA data: *, *p* < 0.05; **, *p* < 0.01; ***, *p* < 0.001; ****, *p* < 0.0001; For the activity data: §, *p* < 0.05; §§, *p* < 0.01; §§§, *p* < 0.001; §§§§, *p* < 0.0001).

**FIGURE 4:**
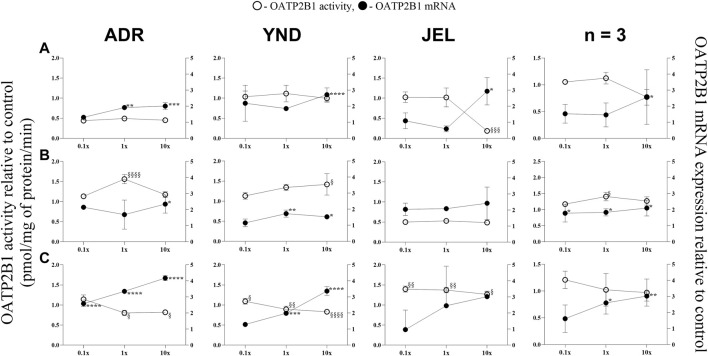
Effect (relative to control) of pregnancy-related hormone (PRH) cocktails on OATP2B1 activity (empty circles; left axis) and mRNA expression (filled circles, right axis) in each of the 3 lots of premenopausal plated human hepatocytes (ADR, YND, and JEL) as well as when these data were pooled (n = 3). PRH cocktails were designed to mimic their *in vivo* plasma concentrations observed in the first **(A)**, second **(B)**, and third **(C)** trimesters of pregnancy. Within each trimester, the effect of the ≈unbound (0.1×), total (1×), and supraphysiological (10×) plasma concentrations of the PRH was studied. Data are mean ± SD of 3 replicates for individual lots and mean ± SD of three lots when pooled. Significance was determined using one-way (within lot) or repeated measures (when data from 3 lots were pooled) ANOVA with Dunnett’s correction. For the mRNA data: *, *p* < 0.05; **, *p* < 0.01; ***, *p* < 0.001; ****, *p* < 0.0001; For the activity data: §, *p* < 0.05; §§, *p* < 0.01; §§§, *p* < 0.001; §§§§, *p* < 0.0001).

**FIGURE 5:**
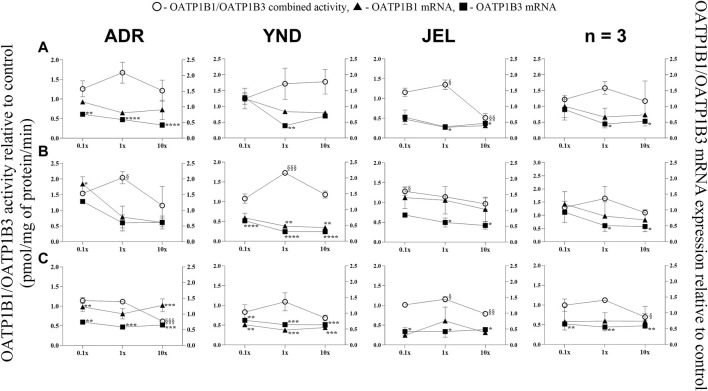
Effect (relative to control) of pregnancy-related hormone (PRH) cocktails on OATP1B1/1B3 activity (empty circles; left axis) and OATP1B1 (filled triangules, right axis) and OATP1B3 (filled squares; right axis) mRNA expression in each of 3 lots of premenopausal plated human hepatocytes (ADR, YND, and JEL) as well as when data were pooled (n = 3). PRH cocktails were designed to mimic their *in vivo* plasma concentrations observed in the first **(A)**, second **(B)**, and third **(C)** trimesters of pregnancy. Within each trimester, the effect of the ≈unbound (0.1×), total (1×), and supraphysiological (10×) plasma concentrations of the PRH was studied. Data are mean ± SD of 3 replicates for individual lots and mean ± SD of three lots when pooled. Significance was determined using one-way (within lot) or repeated measures (when data from 3 lots were pooled) ANOVA with Dunnett’s correction. For the mRNA data: *, *p* < 0.05; **, *p* < 0.01; ***, *p* < 0.001; ****, *p* < 0.0001; For the activity data: §, *p* < 0.05; §§, *p* < 0.01; §§§, *p* < 0.001; §§§§, *p* < 0.0001).

Influx transporter mRNA expression and activity demonstrated significant inter-lot variability after PRH cocktail exposure (Figures 1–5). For this reason, our analysis and narrative below focused on the data obtained in the individual lots rather than that observed when data from all three lots were pooled (see discussion for details). In contrast, CYP3A4 mRNA induction by rifampin (10 µM) showed much less inter-lot variability (Table 4). In the narrative below, we have emphasized the results obtained with the PRH 1x cocktails as these are likely to be more relevant for the *in vivo* situation for reasons discussed before (Zhang et al., 2015). Briefly, based on our previous data, we postulate that the 1x cocktails (rather than the 0.1× cocktails) better translate to the *in vivo* situation. These data showed that incubation of PHH with 1x cortisol concentration, but not 0.1x cortisol concentration, induced CYP3A activity comparable to that observed *in vivo* in pregnant people (Zhang et al., 2015).

**TABLE 4:** Induction of CYP3A4 mRNA expression (relative to vehicle control) when PHH (lot ADR, YND, and JEL) were incubated with rifampin (10 µM) for 72 h.

	Relative to control^a^	p-value
ADR	29.84 ± 7.23	0.004
YND	43.34 ± 14.77	0.015
JEL	30.19 ± 15.56	0.054
n = 3^b^	34.53 ± 6.39^b^	0.017

^a^ Data shown are mean ± SD, of triplicates.

^b^ Data are mean ± SD, of the three lots. p-values were estimated using the unpaired *t*-test (within lot) or paired *t*-test (all 3 lots).

In general, our data (Figures 1–5; Table 5) showed that the PRH cocktails a) significantly induced mRNA expression in the order OAT2 ≈ NTCP ≈ OCT1 > OATP2B1 and repressed mRNA expression in the order OATP1B3 > OATP1B1; b) these changes translated into significant induction of OAT2 (T1-T3) and NTCP (T2-T3, in only 2 lots) activity at 1x PRH concentration. The change in mRNA expression mostly exceeded the change in transporter activity; c) the effect of PRH on both the mRNA expression and activity within each trimester was mostly concentration-dependent (0.1×, 1×, or 10×) and in some cases approached or attained a plateau (e.g., OAT2 in lot ADR in all trimesters). In contrast, the increase in PRH concentrations from T1 to T3 did not result in a consistent increase in transporter activity; d) compared with the influx transporters, the change in mRNA expression of the efflux transporters was much more modest; MRP2 and BSEP gene expression was induced the most (Supplementary Figures S1–6).

**TABLE 5:** Transporter activity or mRNA expression (expressed as percent of control) significantly induced or repressed by T1-T3 PRH 1x cocktails in 3 lots of PHH.

		T1*		T2*		T3*	
		mRNA	Activity	mRNA	Activity	mRNA	Activity
OAT2	ADR	↑389%	↑656%	↔	↑527%	↑1247%	↑771%
	YND	↑1086%	↑369%	↑950%	↑443%	↑485%	↑517%
	JEL	↔	↔	↑299.33%	↑94.08%	↔	↔
NTCP	ADR	↑404%	↔	↔	↑797%	↑554%	↑119%
	YND	↑452%	↔	↑531%	↔	↑503%	↑320%
	JEL	↔	↔	↑642%	↑942%	↔	↑138%
OCT1	ADR	↑247%	↔	↑306%	↑85%	↑500%	↔
	YND	↑303%	↔	↑716%	↑51%	↑552%	↔
	JEL	↔	↔	↑498%	↑71%	↔	↔
OATP2B1	ADR	↑92%	↔	↔	↑56%	↑235%	↓20%
	YND	↔	↔	↑72%	↔	↑98%	↓10%
	JEL	↔	↔	↔	↔	↔	↑37%
OATP1B1	ADR	↔	↔	↔	↑104%	↔	↔
	YND	↔	↔	↓52%	↑73%	↓53%	↔
	JEL	↔	↑35%	↔	↔	↔	↑16%
OATP1B3	ADR	↓41%	↔	↔	↑104%	↓41%	↔
	YND	↓61%	↔	↓70%	↑73%	↓36%	↔
	JEL	↓65%	↑35%	↓40%	↔	↓58%	↑16%

^*^T1, T2 and T3: First, second and third trimester of pregnancy. Values shown are percent significantly downregulated (↓), upregulated (↑), or not changed (↔) relative to the control treatment. The activity data for transporters OATP1B1 and OATP1B3 represents their combined activity.

### 3.1 OAT2 and NTCP activity and mRNA expression were significantly induced by the PRH cocktails in premenopausal PHH

OAT2 mRNA expression was significantly induced in two lots (ADR and YND) by T1-T3 1x and/or 10× PRH cocktails (Figure 1), except in lot ADR (T2 cocktails; Figure 1B). In contrast, the third lot (JEL) demonstrated significant mRNA induction with only the T2 cocktails (Figure 1B). Interestingly, in one lot (YND) mRNA induction was greater with the T1 compared to the T3 cocktails. Induction of OAT2 activity aligned with induction of mRNA expression and was significant for T1-T3 1x PRH cocktail in only two lots (ADR and YND) (Figure 1). When data from all three lots were combined, only the transporter mRNA expression (and not its activity) was significantly induced by both the T2 and T3 1× PRH cocktails (Figures 1B,C).

Similarly, NTCP mRNA expression showed significant induction by the 1× cocktails for most trimesters in only two lots (ADR and YND) (Figure 2). When data from all three lots were pooled, NTCP mRNA expression was significantly induced by only T1 and T2 10x PRH cocktails (Figures 2A,B). Generally, the trend in changes in NTCP activity followed those of changes in mRNA expression (Figure 2). Unlike OAT2, NTCP activity was induced in all three lots by the T3 1× cocktail. However, when the data were pooled, NTCP activity was not induced at any of the concentrations or trimesters.

### 3.2 The mRNA expression (but not activity) of OCT1 and OATP2B1 was induced by the PRH cocktails in premenopausal PHH

OCT1 mRNA expression was significantly induced in three lots across all trimesters by the 1× and/or 10× PRH cocktails (Figure 3), except in one lot (JEL) with the T3 cocktails (Figure 3C). When data from all three lots were combined, OCT1 mRNA expression was significantly induced by the T2 and T3 1x PRH cocktails and by T1-T3 10× PRH cocktails (Figure 3). OCT1 activity was induced in all three lots by only T2 1× PRH cocktail (Figure 3B) and in two lots (YND and JEL) by T1 10x PRH cocktail (Figure 3A). Repression of OCT1 activity was significant in one lot (ADR) by T1 10× PRH cocktail (Figure 3A). When the data were pooled, OCT1 activity was significantly induced by only T1 and T2 1× and 10× (T1 only) PRH cocktails (Figure 3).

Similarly, OATP2B1 mRNA expression was significantly induced in the three lots by the T1-T3 1× and/or 10× PRH cocktails (Figure 4), except in one lot (JEL) at T2 cocktails (Figure 4B). When data from all three lots were combined, OATP2B1 mRNA expression was significantly induced by T1-T3 1× and/or 10× cocktails (Figure 4). However, the trend in changes in OATP2B1 activity did not align with those of changes in mRNA expression (Figure 4). OATP2B1 activity was modestly but significantly repressed in two lots (ADR and YND) by T3 1× and 10× PRH cocktails and in one lot (JEL) by T1 10× PRH cocktail. When data were pooled, OATP2B1 activity was modestly induced by only T2 1× PRH cocktail (Figure 4B).

### 3.3 OATP1B1 and 1B3 mRNA expression (but not activity) was repressed by the PRH cocktails in premenopausal PHH

OATP1B1 mRNA expression showed significant repression in only one lot (YND) for T2 (1× and 10× cocktails) and T3 at 0.1×, 1×, and 10× cocktails (Figure 5). Conversely, significant repression of OATP1B3 mRNA expression occurred at 1× and/or 10× cocktails for all trimesters and lots, except ADR for all T2 cocktails. Additionally, when data were pooled, only OATP1B3 mRNA repression was significant at 1x and 10x for all trimester PRH cocktails. Since selective substrates of OATP1B1 or OATP1B3 are not available, their combined activity was measured. This combined activity was modestly but significantly induced in two lots (ADR and YND) by the T2 1× PRH cocktail, and in one lot (JEL) by T1 1× and T2 0.1× PRH cocktails. Significant repression of the combined OATP1B1/1B3 activity was observed in 2 lots (ADR and JEL) by only the T3 10× PRH cocktail (Figure 5C). When data from all three lots were pooled, the combined OATP1B1/1B3 activity was repressed by only T3 10× PRH cocktail (Figure 5C).

### 3.4 mRNA expression of efflux transporter was more modestly affected by the PRH cocktails than that of the influx transporters in premenopausal PHH

Compared with the influx transporters, induction of mRNA expression of the efflux transporters by the PRH cocktails was modest (Supplementary Figures S1–S6). mRNA expression of MRP2 and BSEP was the most significantly induced (Supplementary Figures S1, S2). MRP2 (Supplementary Figure S1) and MRP3 (Supplementary Figure S3) showed induction (with differing PRH cocktails) across all three lots, while BCRP showed modest induction in 2 lots (ADR and YND) with only some PRH cocktails (Supplementary Figure S6). P-gp and MRP4 mRNA expression was significantly repressed at the 10× T3 concentration in one lot (JEL) (Supplementary Figures S4, S5).

## 4 Discussion

To date, this study represents the first comprehensive investigation of changes in hepatic influx transporter activity and mRNA expression produced by PRH cocktails. The design of our study incorporates features that are novel and not employed by other researchers in this field (Khatri et al., 2021a; Khatri et al., 2021b; Fashe et al., 2022; Alshabi et al., 2023). First, we investigated the effect of a cocktail of PRHs on both mRNA expression and activity of influx transporters while others have investigated their effect on only the protein expression of these transporters (Fashe et al., 2022). Second, our PRH cocktails included more pregnancy hormones (testosterone, oxytocin) than those employed by others (Khatri et al., 2021a; Khatri et al., 2021b; Fashe et al., 2022; Alshabi et al., 2023). Third, the PRH cocktails we used were designed to mimic the *in vivo* plasma concentrations of the PRH in the three trimesters of pregnancy (Table 2). Within each trimester, the effects of the PRH on the activity and/or mRNA expression of hepatic transporters were studied at their approximate unbound (0.1×), total (1×), and supraphysiological (10x) plasma concentrations (Table 3). Fourth, since hepatic media traditionally contain corticosteroids (e.g., cortisol or dexamethasone), we deliberately utilized a custom corticosteroid-free media for culturing our PHH. This circumvents the potential confounding effects of these steroids, already present in the media, on hepatic transporter expression and activity when incubated with the PRH cocktails (which also contain cortisol). Fifth, we adjusted our PRH cocktail concentrations based on their depletion by the PHH. Sixth, we used premenopausal PHH as their response to PRH cocktails may differ from hepatocytes obtained from postmenopausal women or men. Finally, as an internal biological quality control, we quantified the induction of CYP3A4 mRNA by rifampin (10 µM) in each PHH experiment.

In general, the PRH cocktails induced mRNA expression of influx transporters to a greater extent than that of the efflux transporters. For this reason, as well as the fact that efflux transporter activity is best studied using sandwich-cultured hepatocytes, the effect of PRH cocktail on only the activity of the influx transporters was quantified.

While we conducted studies within each trimester at 0.1×, 1×, and 10× PRH cocktails, we believe that the results obtained with the 1× cocktails (Table 5) are likely more relevant to the *in vivo* situation, as discussed before (Zhang et al., 2015). At this concentration, PRH (T1, T2, and/or T3) cocktails significantly induced (OAT2 ≈ NTCP ≈ OCT1 > OATP2B1) or repressed (OATP1B3) activity and/or mRNA expression in at least two of the three lots of PHH (Table 5). Among these transporters, at the concentrations relevant to *in vivo* (1x cocktails), PRH significantly induced the activity of only OAT2 (T1-T3) and NTCP (T2, T3) in at least two lots of PHH (Table 5). In general, lot ADR and YND behaved similarly, while lot JEL (African American) was the odd one out. Due to this significant inter-lot variability, these effects were mostly insignificant when data for all three lots were pooled (Figures 1–5). However, this inter-lot variability *in vitro* (Table 5) likely reflects inter-subject variability *in vivo*. Thus, it is possible that many (but not all) pregnant people may demonstrate significant upregulation in activity of OAT2 and NTCP. The mechanistic basis for this inter-lot variability needs further investigation.

Of the influx transporters, OAT2 and NTCP were found to be the two most induced transporters (activity and mRNA expression). In contrast, sandwich-cultured hepatocytes exposed to a comparable PRH cocktail showed reduced or no change in OAT2 protein expression (activity was not measured) relative to control (Fashe et al., 2022). We speculate that this difference might be due to differences in the composition of PRH cocktails (they did not include testosterone and oxytocin) and the sandwich-cultured model employed by the study. OAT2 is expressed in both the liver and the kidneys and can transport a wide array of clinically important drugs such as antibiotics, antivirals, and chemotherapy agents, plus endogenous compounds such as cGMP, uric acid, creatinine, and prostaglandins (Kimoto et al., 2018; Özvegy-Laczka et al., 2023). Some well-characterized drug substrates of the hepatic OAT2 (Kimoto et al., 2018), commonly used during pregnancy, include the non-steroidal anti-inflammatory ibuprofen (McKenna and McIntyre, 2006; Cejvanovic et al., 2014), the sulfonylurea gliclazide (Kelty et al., 2020) and the antibiotic sulfamethoxazole (Muanda et al., 2018). If the effect of PRH on the induction of OAT2 activity is replicated *in vivo*, this enhanced OAT2 activity could lead to elevated peak hepatic drug concentrations, potentially resulting in hepatic toxicity if toxicity is correlated with peak hepatic concentrations. Interestingly, as discussed by Patilea-Vrana and Unadkat (Patilea-Vrana and Unadkat, 2016), if OAT2 is the rate-determining step for the clearance of the drug and renal clearance is negligible, the hepatic drug AUC (and therefore toxicity and efficacy, if correlated with the AUC) will not be affected. Since hepatic OAT2 has a narrow spectrum of known drug substrates, and since the PK of these drugs has not been studied in pregnancy, it will be important to determine if these findings translate to *in vivo*. It is possible that they do so as renal OAT2 activity is induced during pregnancy (Peng et al., 2021).

NTCP, located exclusively on the sinusoidal membrane of hepatocytes, plays a crucial role in bile acid influx. Many statins are also substrates of NTCP, notably rosuvastatin (Kumar et al., 2019; Kumar et al., 2021; Storelli et al., 2022) and pitavastatin (Yin et al., 2024). Thus, induction of NTCP could result in enhanced clearance of these drugs during pregnancy. NTCP activity may be induced to clear the elevated conjugated bile acids plasma concentrations during pregnancy (Gagnon et al., 2021) as a protective mechanism to avoid bile acid unbalance during pregnancy. For example, taurocholic acid plasma concentrations are elevated during pregnancy (Czuba et al., 2023). No change in NTCP protein was observed after exposure of sandwich-cultured human hepatocytes to PRH cocktails (composition different from ours, see above) (Fashe et al., 2022). Interestingly, prolactin, placental lactogen and growth hormone induced NTCP expression in rat hepatocytes (Cao et al., 2001). Also, corticosteroid treatment increases NTCP expression in mice (Rose et al., 2011). However, other animal studies suggest that pregnancy represses NTCP mRNA and protein expression (Arrese et al., 2003; Abu-Hayyeh et al., 2010; Aleksunes et al., 2012).

OCT1 transports metformin into the liver to produce its antidiabetic effect including in pregnant people (Newman et al., 2023). While OCT1 is predominantly expressed in the liver (Zeng et al., 2023), OATP2B1 exhibits a broader tissue distribution, such as in the liver, brain, small intestine and colon, kidney, and placenta (McFeely et al., 2019; Kinzi et al., 2021) reflecting the diverse regulatory mechanisms at play. In general, PRH resulted in the induction of mRNA expression of OCT1 and OATP2B1 without a consistent concurrent change in their activity (Figures 3, 4). This mRNA and activity incongruence suggests intricate regulatory pathways governing transporter function beyond transcriptional control. Indeed, the regulation of OCT1 and OATP2B1 involves multiple mechanisms, including the activation of nuclear receptors and post-translational modifications (Brouwer et al., 2022). For OATP2B1, the regulation also involves post-translational internalization and miRNA-mediated processes (Brouwer et al., 2022). Whereas OCT1 regulation encompasses methylation and kinase-mediated tyrosine phosphorylation (Brouwer et al., 2022). These findings highlight the intricate interplay of various regulatory mechanisms in modulating transporter activity, contributing to the nuanced response observed in our study.

Conversely, OATP1B1/1B3 mRNA expression and their combined activity were repressed by only the T3 10× PRH cocktail (Figure 5C, pooled data). These transporters are important in the hepatic uptake and clearance of statins and several other drugs including glyburide (Yin et al., 2024). Glyburide is widely used during pregnancy to treat diabetes (Affres et al., 2021). In agreement with our findings, OATP activity appears to be reduced during pregnancy as evidenced by a significantly greater area under the plasma concentration-time profile of rosuvastatin antenatal vs. *postpartum* when the same women were dosed during these two periods (Moreira et al., 2023). Again, sandwich-cultured hepatocytes exposed to PRH cocktails (composition different from ours) did not show significant changes in OATP1B1/1B3 protein expressions across the second and third trimesters of pregnancy (Fashe et al., 2022). Moreover, no changes in OATP1B1/1B3 protein abundance were observed in liver-derived small extracellular vesicles obtained from the sera of pregnant women (n = 3) throughout the trimesters of pregnancy (Rodrigues et al., 2021). Of note, measuring protein abundance may be less sensitive (due to the variability in the proteomics assay) than measuring activity. These data warrant further investigations into hepatic OATP activity and factors that regulate OATP activity during pregnancy.

Limitations of this study include the small sample size and the considerable inter-lot variability as well as our inability to determine efflux transporter activity. The use of PRH cocktails, while they replicate *in vivo* PRH concentrations, limited our ability to identify the hormone responsible for the observed changes in transporter mRNA expression and activity. Besides posttranslational processes, some of the discrepancies between the effect on mRNA expression and transporter activity may be due to the long half-lives of the transport proteins. Unfortunately, the *in vivo* half-life of many transporter proteins is unknown. If these half-lives are very long, incubation of PHH for 72 h may have been insufficient for the protein activity to reach a new steady-state after enhanced transcription of the corresponding gene by the PRH. Also, it is possible that endogenous factors (e.g., changes in maternal microbiome), other than the PRH, are the cause of any *in vivo* changes in transporter activity during pregnancy.

## 5 Conclusion

This study represents a comprehensive investigation of the impact of PRH, at plasma concentrations observed at all stages of pregnancy, on the activity and mRNA expression of multiple hepatic transporters in premenopausal PHH. The major findings of this study were that significant inter-lot variability was observed in mRNA expression and activity when PHH were exposed to PRH. In general, PRH significantly induced (OAT2 ≈ NTCP ≈ OCT1 > OATP2B1) activity and/or mRNA expression in PHH while they repressed OATP1B3 > OATP1B1 mRNA expression which did not always result in repression of activity. Of these transporters, at the *in vivo* relevant concentrations (1× cocktails), the PRH significantly induced the activity of only OAT2 (T1-T3) and NTCP (T2, T3) in at least 2 lots of PHH (Table 5). The ultimate goal of our study was to generate data that can be used to populate physiologically based pharmacokinetic models (PBPK) to predict changes in *in vivo* transporter-mediated clearance of drugs throughout pregnancy. To do so, the data presented here need to be verified through *in vivo* probe PK studies during pregnancy as we have done before (Hebert et al., 2008). Our findings also provide a foundation for studies to elucidate the mechanisms by which PRH regulate the affected transporters.

## Data Availability

The raw data supporting the conclusions of this article will be made available by the authors, without undue reservation.
