# Supplementary material for: The effect of pregnancy-related hormones on hepatic transporters: studies with premenopausal human hepatocytes
Source: Front Pharmacol. 2024 Aug 7;15:1440010. doi: 10.3389/fphar.2024.1440010 (PMC11335556; doi:10.3389/fphar.2024.1440010)
Supplement: Supplementary file 1 [file Image1.pdf]

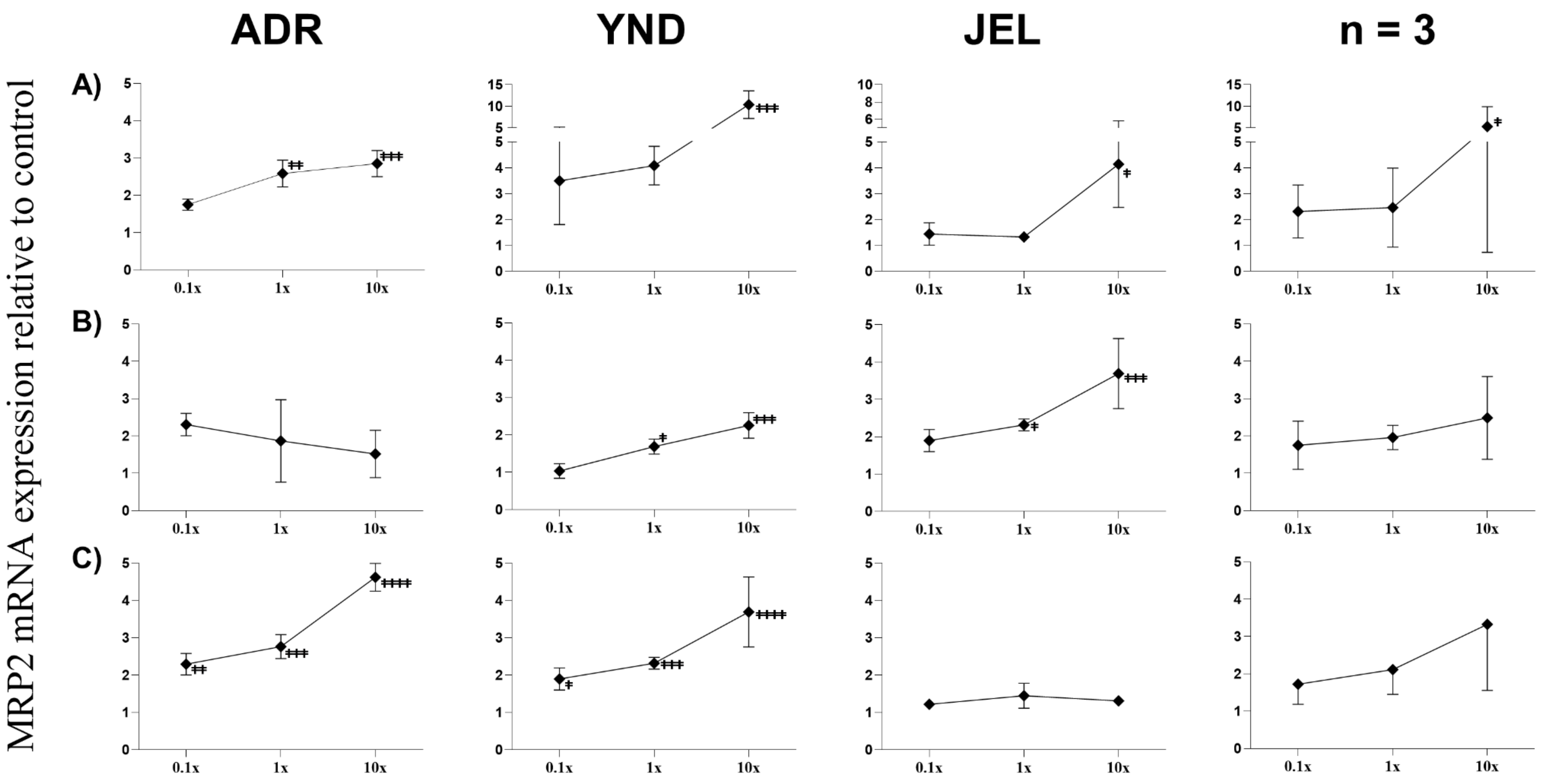

Supplementary Figure 1. Effect (relative to control) of pregnancy-related hormone (PRH) cocktails on MRP2 mRNA expression (black diamonds) in each of the 3 lots of premenopausal plated human hepatocytes (ADR, YND, and JEL) as well as when data were pooled (n = 3). PRH cocktails were designed to mimic their *in vivo* plasma concentration observed in the first (A), second (B), and third (C) trimesters of pregnancy. Within each trimester, the effect of the ≈unbound (0.1x), total (1x), and supraphysiological (10x) plasma concentrations of the PRH was studied. Data are mean ± SD of 3 replicates for individual lots and mean ± SD of three lots when pooled. Significance was determined using one-way (within a lot) or repeated measures (when data from 3 lots were pooled) ANOVA with Dunnett's correction. ǂ, $p<0.05$; ǂǂ, $p<0.01$; ǂǂǂ, $p<0.001$; ǂǂǂǂ, $p<0.0001$.

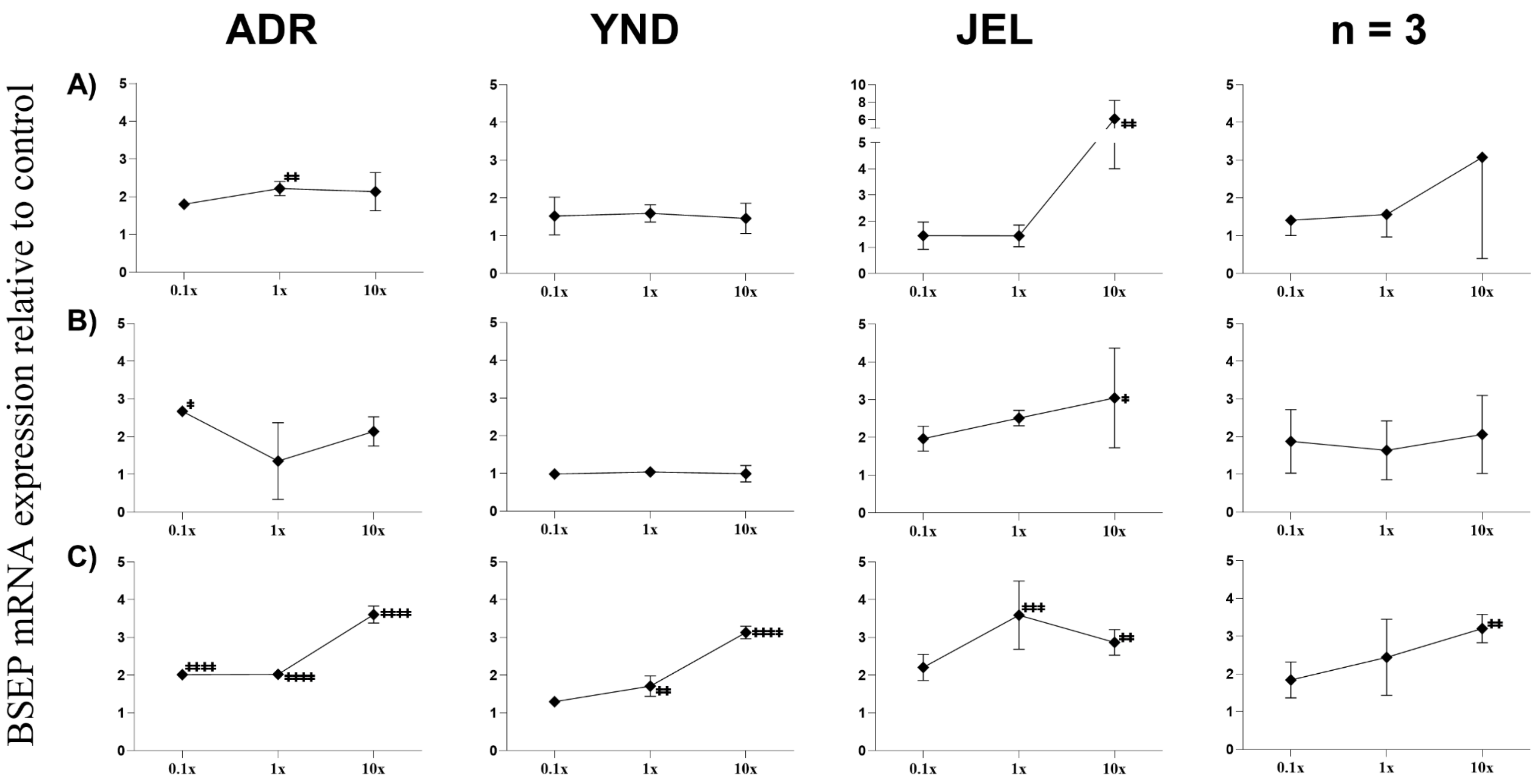

Supplementary Figure 2. Effect (relative to control) of pregnancy-related hormone (PRH) cocktails on BSEP mRNA expression (black diamonds) in each of the 3 lots of premenopausal plated human hepatocytes (ADR, YND, and JEL) as well as when data were pooled (n = 3). PRH cocktails were designed to mimic their *in vivo* plasma concentration observed in the first (A), second (B), and third (C) trimesters of pregnancy. Within each trimester, the effect of the ≈unbound (0.1x), total (1x), and supraphysiological (10x) plasma concentrations of the PRH was studied. Data are mean $\pm$ SD of 3 replicates for individual lots and mean $\pm$ SD of three lots when pooled. Significance was determined using one-way (within a lot) or repeated measures (when data from 3 lots were pooled) ANOVA with Dunnett's correction. ǂ, $p<0.05$; ǂǂ, $p<0.01$; ǂǂǂ, $p<0.001$; ǂǂǂǂ, $p<0.0001$.

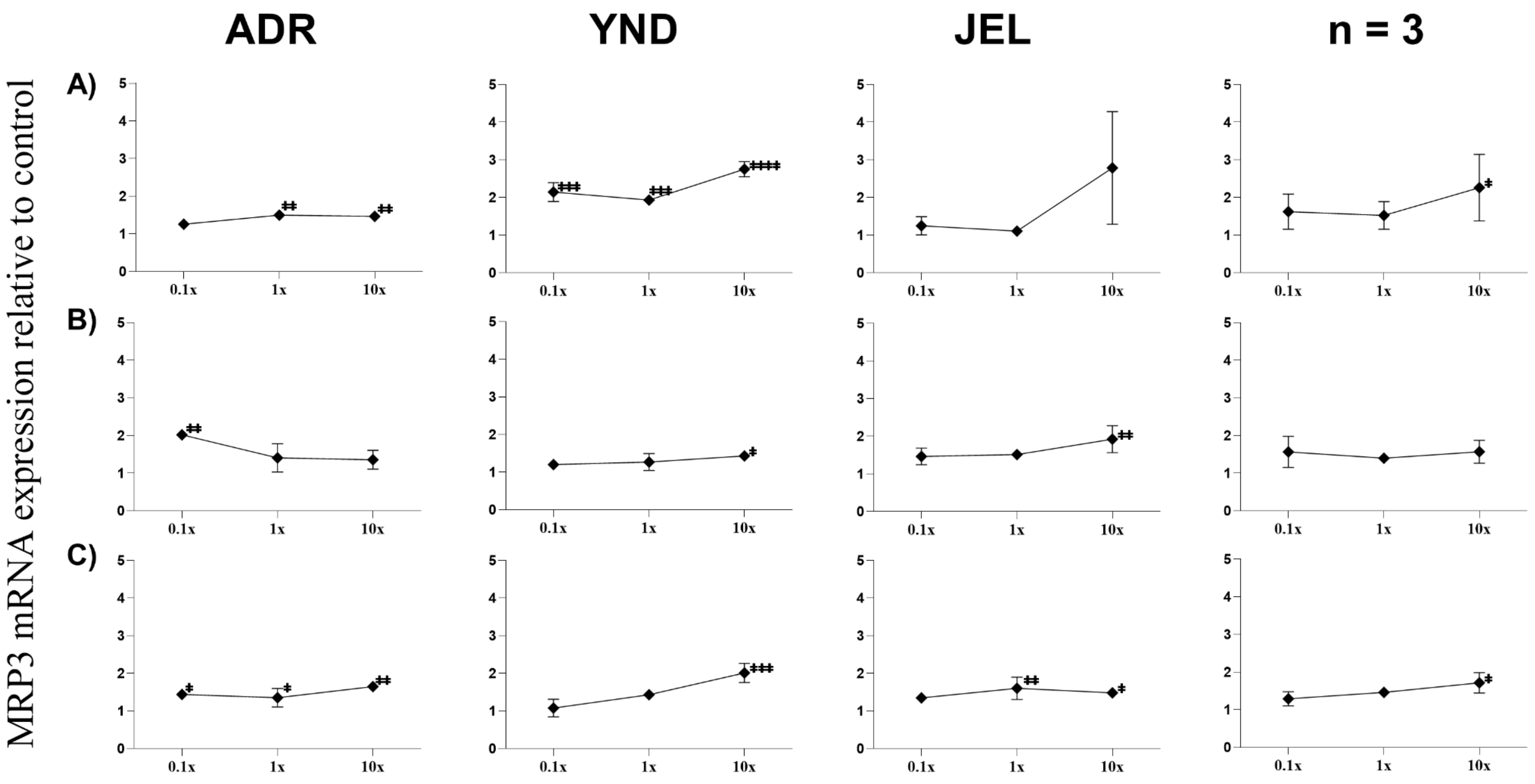

Supplementary Figure 3. Effect (relative to control) of pregnancy-related hormone (PRH) cocktails on MRP3 mRNA expression (black diamonds) in each of the 3 lots of premenopausal plated human hepatocytes (ADR, YND, and JEL) as well as when data were pooled (n = 3). PRH cocktails were designed to mimic their *in vivo* plasma concentration observed in the first (A), second (B), and third (C) trimesters of pregnancy. Within each trimester, the effect of the ≈unbound (0.1x), total (1x), and supraphysiological (10x) plasma concentrations of the PRH was studied. Data are mean ± SD of 3 replicates for individual lots and mean ± SD of three lots when pooled. Significance was determined using one-way (within a lot) or repeated measures (when data from 3 lots were pooled) ANOVA with Dunnett's correction. ǂ, $p<0.05$; ǂǂ, $p<0.01$; ǂǂǂ, $p<0.001$; ǂǂǂǂ, $p<0.0001$.

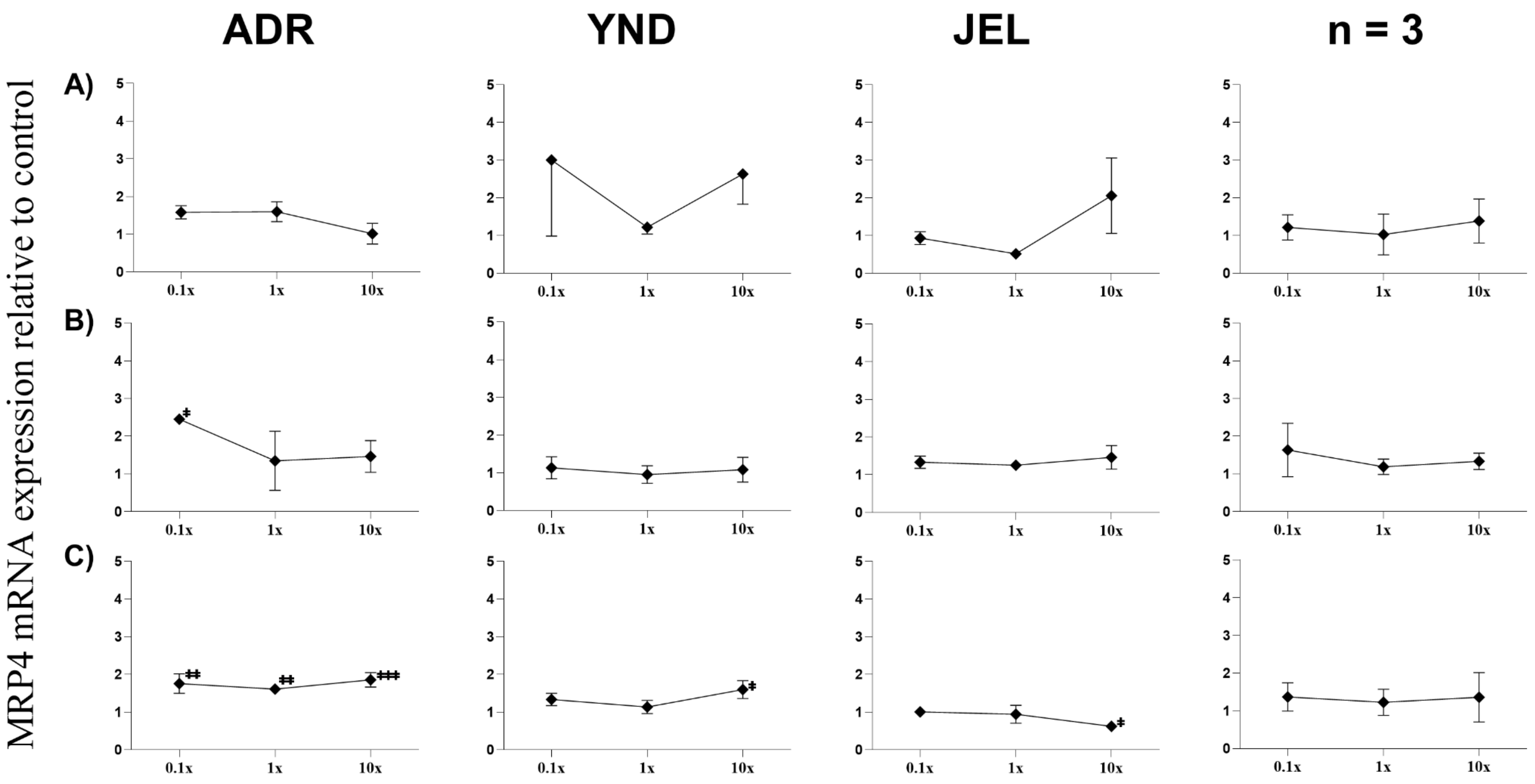

Supplementary Figure 4. Effect (relative to control) of pregnancy-related hormone (PRH) cocktails on MRP4 mRNA expression (black diamonds) in each of the 3 lots of premenopausal plated human hepatocytes (ADR, YND, and JEL) as well as when data were pooled (n = 3). PRH cocktails were designed to mimic their *in vivo* plasma concentration observed in the first (A), second (B), and third (C) trimesters of pregnancy. Within each trimester, the effect of the ≈unbound (0.1x), total (1x), and supraphysiological (10x) plasma concentrations of the PRH was studied. Data are mean ± SD of 3 replicates for individual lots and mean ± SD of three lots when pooled. Significance was determined using one-way (within a lot) or repeated measures (when data from 3 lots were pooled) ANOVA with Dunnett's correction. ǂ, $p<0.05$; ǂǂ, $p<0.01$; ǂǂǂ, $p<0.001$; ǂǂǂǂ, $p<0.0001$.

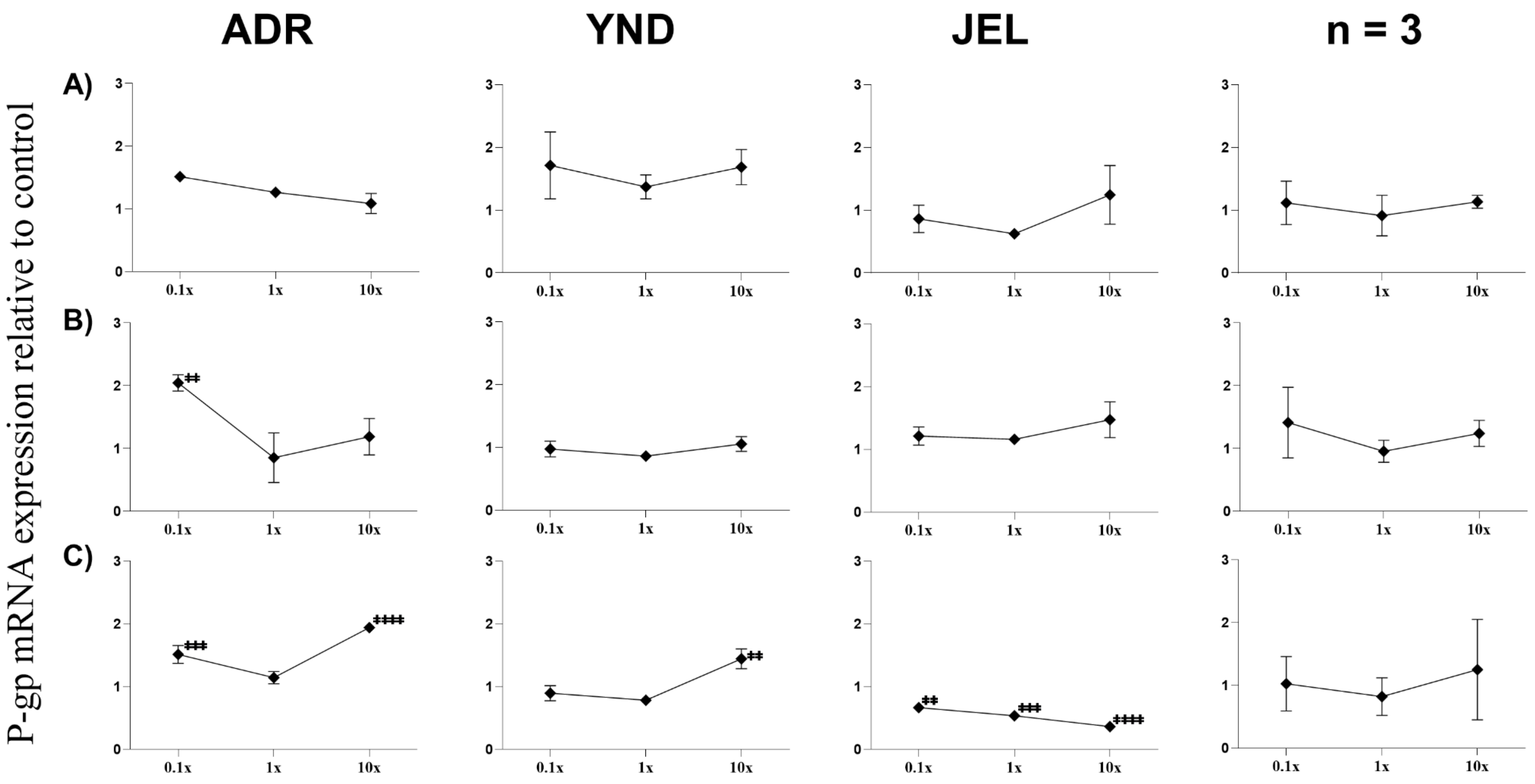

Supplementary Figure 5. Effect (relative to control) of pregnancy-related hormone (PRH) cocktails on P-gp mRNA expression (black diamonds) in each of three 3 lots of premenopausal plated human hepatocytes (ADR, YND, and JEL) as well as when data were pooled (n = 3). PRH cocktails were designed to mimic their *in vivo* plasma concentration observed in the first (A), second (B), and third (C) trimesters of pregnancy. Within each trimester, the effect of the ≈unbound (0.1x), total (1x), and supraphysiological (10x) plasma concentrations of the PRH was studied. Data are mean ± SD of 3 replicates for individual lots and mean ± SD of three lots when pooled. Significance was determined using one-way (within a lot) or repeated measures (when data from 3 lots were pooled) ANOVA with Dunnett's correction. ǂ, $p<0.05$; ǂǂ, $p<0.01$; ǂǂǂ, $p<0.001$; ǂǂǂǂ, $p<0.0001$.

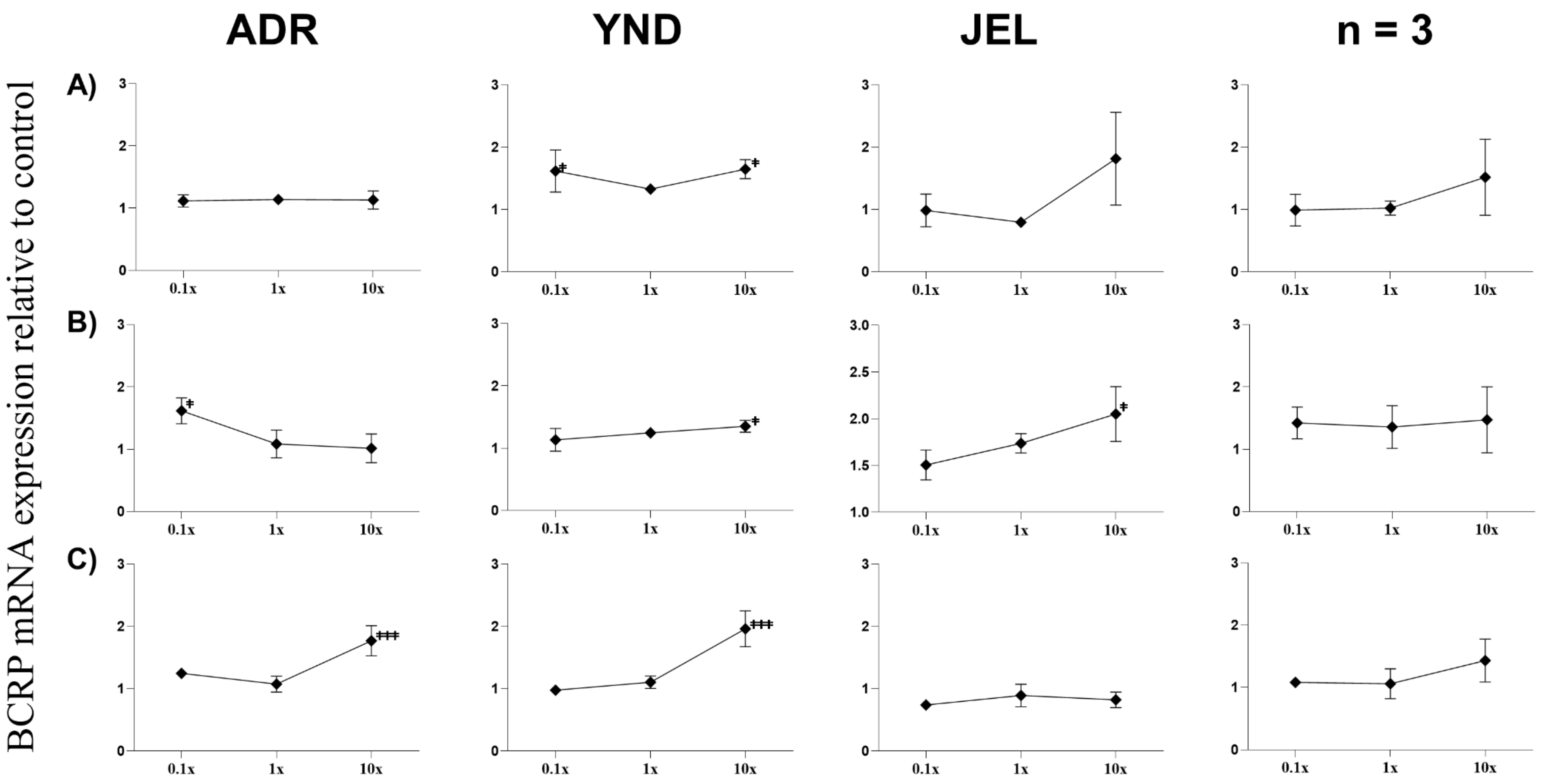

Supplementary Figure 6. Effect (relative to control) of pregnancy-related hormone (PRH) cocktails on BCRP mRNA expression (black diamonds) in each of the 3 lots of premenopausal plated human hepatocytes (ADR, YND, and JEL) as well as when data were pooled (n = 3). PRH cocktails were designed to mimic their *in vivo* plasma concentration observed in the first (A), second (B), and third (C) trimesters of pregnancy. Within each trimester, the effect of the ≈unbound (0.1x), total (1x), and supraphysiological (10x) plasma concentrations of the PRH was studied. Data are mean ± SD of 3 replicates for individual lots and mean ± SD of three lots when pooled. Significance was determined using one-way (within a lot) or repeated measures (when data from 3 lots were pooled) ANOVA with Dunnett's correction. ǂ, $p<0.05$; ǂǂ, $p<0.01$; ǂǂǂ, $p<0.001$; ǂǂǂǂ, $p<0.0001$.
